# The 5th ISCB Wikipedia Competition: Coming to a Classroom Near You?

**DOI:** 10.1371/journal.pcbi.1005235

**Published:** 2016-12-29

**Authors:** Alastair M. Kilpatrick

**Affiliations:** Department of Pediatrics, University of California, San Diego, La Jolla, California, United States of America

The International Society for Computational Biology (ISCB) is pleased to announce the 5th ISCB Wikipedia Competition. The competition has been run annually since 2012 and awards students and trainees for the best contributions to computational biology-related articles [1]. ISCB runs the competition in collaboration with WikiProject Computational Biology, a group of around 130 editors overseeing the roughly 1,300 computational biology-related articles on Wikipedia. Entry to the competition is open internationally to students and trainees of any level, both as individuals and as groups. Further information about the competition can be found here: https://en.wikipedia.org/wiki/WP:ISCB2016.

The 2016 competition began on 13 July 2016, coinciding with the Intelligent Systems for Molecular Biology (ISMB) conference, and will end on 31 December 2016. Each article entered in the competition will be reviewed by students nominated by the ISCB Student Council, and a shortlist of entries will be examined by a judging panel. The winners will be presented with their awards at ISMB/ECCB 2017 in Prague.

As of the 2015 competition, entries in languages other than English are welcomed; the 2015 competition saw the creation and expansion of several articles in Spanish, and the newly-created Spanish Wikipedia entry for BioJava was awarded second prize. The 2015 competition was also the first to include a Wikidata component. Similar to Wikipedia, Wikidata is a collaboratively edited knowledge base, intended to provide a common source of data to be used primarily by Wikipedia but also the general public. Launched in 2012, Wikidata plays an increasingly important role in communicating all types of science to the public, and the inclusion of a Wikidata component in the competition aims to improve data content related to computational biology. In the 2015 competition, prizes were awarded for contributions to the Vienna RNA Package and Docking Wikidata items. We hope to see an increased number of contributions in other languages and to Wikidata content in this year's competition.

As the 2016 competition begins, the ISCB and WikiProject Computational Biology remain keen to grow the quality and depth of computational biology coverage over all Wikimedia projects and wish to encourage the widest possible range of students and trainees to participate. We particularly encourage teachers, tutors, and lecturers to use the competition in their courses, with contributions to relevant Wikipedia or Wikidata content as part of a class assignment. Wikipedia provides information and resources for running editing projects as part of school and university classes (see https://en.wikipedia.org/wiki/WP:SUP), and *PLOS Computational Biology* has also published tips for Wikipedia beginners [2].

As described previously, unlike traditional class assignments, contributing to Wikipedia means that students' work will become publicly available to future researchers. Several courses already contain such a component; a 2015 course on Biological Clocks at Washington University in St. Louis, Missouri added a significant amount of information on the topic of chronobiology, including new biography articles on notable researchers in the field. Besides the primary contribution to public knowledge, the competition also provides the opportunity to develop effective communication skills with a range of audiences and the ability to function effectively as part of a team for group entries, two of ISCB's core bioinformatics competencies [3].

We hope that the ISCB Wikipedia Competition will continue to grow and make an increasing contribution to both the quality of computational biology information freely available online and the training of the next generation of computational biologists.
